# Maximizing Early Dementia Detection Through Medicare Annual Wellness Visits

**DOI:** 10.1001/jamanetworkopen.2024.37162

**Published:** 2024-10-01

**Authors:** Márlon Juliano Romero Aliberti, Thiago J. Avelino-Silva, Claudia Kimie Suemoto

**Affiliations:** Laboratorio de Investigacao Medica em Envelhecimento (LIM-66), Servico de Geriatria, Hospital das Clinicas HCFMUSP, Faculdade de Medicina, Universidade de Sao Paulo, Sao Paulo, Brazil; Research Institute, Hospital Sirio-Libanes, Sao Paulo, Brazil; Laboratorio de Investigacao Medica em Envelhecimento (LIM-66), Servico de Geriatria, Hospital das Clinicas HCFMUSP, Faculdade de Medicina, Universidade de Sao Paulo, Sao Paulo, Brazil; Division of Geriatrics, University of California, San Francisco; Laboratorio de Investigacao Medica em Envelhecimento (LIM-66), Servico de Geriatria, Hospital das Clinicas HCFMUSP, Faculdade de Medicina, Universidade de Sao Paulo, Sao Paulo, Brazil

Early detection of cognitive impairment is a critical public health concern, directly affecting the quality of care and life for millions of older adults. Globally, approximately 57 million people live with dementia, including an estimated 6.7 million US individuals aged 65 years or older with Alzheimer disease.^1^ As life expectancy increases, the prevalence of dementia is expected to double by 2050, emphasizing the urgent need for early detection strategies. The economic burden is substantial, with costs projected to exceed $1 trillion annually by 2030. Despite this, up to 60% of dementia cases go undiagnosed in primary care, highlighting the importance of systematic screening efforts to ensure more cases are identified promptly.^1,2^

Tzeng et al^3^ showed the critical role of annual wellness visits (AWVs) in the early identification of mild cognitive impairment (MCI) and Alzheimer disease and related dementias (ADRD) among Medicare beneficiaries. They analyzed data from over 549 000 Medicare beneficiaries in Texas, revealing that AWVs were associated with a 21% increase in the likelihood of receiving an MCI diagnosis and a 4% increase in the likelihood of receiving an ADRD diagnosis. The study also found that those who received AWVs received a diagnosis of MCI approximately 76 days earlier than those who did not receive AVWs. These findings underscore the importance of AWVs in facilitating early diagnosis and timely management of cognitive impairment in older adults.

The debate over cognitive impairment screening, whether to use a broad or targeted approach, is crucial given the complexities of early dementia detection.^4^ Most dementia cases go undiagnosed, with symptoms often subtle, unreported, or mistaken for normal aging. The US Preventive Services Task Force and the National Institute for Health and Care Excellence both recommend targeted strategies rather than broad screening. Although countries such as South Korea have made significant progress in early brain health screening, others lag behind, underscoring the need for tailored national strategies.^5^ The recent US Food and Drug Administration approval of monoclonal anti-amyloid antibodies for early Alzheimer disease further complicates the landscape, requiring significant changes in care models and infrastructure.^6^ Policies must now support early detection while ensuring safe and equitable access to these complex treatments.

Medicare’s AWVs, introduced under the Patient Protection and Affordable Care Act, provide nearly 60 million beneficiaries with comprehensive health assessments.^7^ These visits typically include a review of medical history, vital signs, risk factors, personalized health advice, and a preventive care plan. A key component is a cognitive assessment, often involving brief standardized tests such as the Mini-Cog, Memory Impairment Screen, or General Practitioner Assessment of Cognition, to detect early signs of impairment. The study by Tzeng et al^3^ offered a comprehensive analysis of how these visits enhance early detection, significantly increasing MCI diagnoses among participants. Early identification is crucial as it allows for proactive management strategies that can slow the progression of MCI to more severe dementia. Patients who receive a diagnosis early can benefit from personalized care plans addressing modifiable risk factors such as hypertension, diabetes, and lifestyle choices. In addition, nonpharmacologic interventions such as structured exercise programs and cognitive stimulation activities can help maintain cognitive function.^4^

Although early dementia diagnosis through AWVs presents clear benefits, it also introduces several challenges that warrant further consideration.^4,5^ One significant concern is the potential strain on health care systems, as widespread early detection could lead to an increase in specialist referrals, diagnostic testing, and ongoing management needs, which may overwhelm existing resources, particularly in areas with already limited access to specialized care. Moreover, the cost effectiveness of such screenings remains a complex issue, as the immediate expenses associated with increased health care utilization must be balanced against the long-term savings from delaying more severe stages of dementia and reducing emergency interventions.^4^ In addition, the psychosocial effect on patients and caregivers after an early diagnosis can be profound, potentially leading to anxiety, depression, and the need for greater emotional and psychological support. These challenges highlight the necessity for a robust, well-coordinated approach that ensures health care systems are equipped to not only detect dementia early but also effectively manage the growing demand for comprehensive care and support services.^4,5^

We cannot overlook other opportunities that come from an early dementia diagnosis. Early detection opens the door for crucial conversations about goals of care and advance care planning, allowing patients and families to make informed decisions about future care preferences and end-of-life care.^4^ It also allows health care professionals to implement strategies that reduce cognitive demands by simplifying routines and optimizing pharmacologic regimens, which minimizes the risk of medication errors and reduces the strain on health care resources. For instance, patients with early-stage cognitive impairment might struggle with complex medication regimens, leading to missed doses and hospitalization. This strategy can be managed through simplified medication plans and caregiver involvement. By identifying cognitive impairment early, health care professionals can refer patients to specialists promptly, ensuring they receive the most appropriate care and support as their condition evolves.^4,5^

Although the study by Tzeng et al^3^ provides valuable insights into the role of AWVs in early dementia detection, certain limitations must be considered. Given the indolent progression of dementia, one concern is the possibility of reverse causality. Patients already experiencing subtle but undiagnosed symptoms of cognitive decline might be more likely to attend one of these AWVs, or caregivers might proactively arrange such appointments for patients with suspected cognitive issues. Although this could lead to the diagnosis of preexisting but unrecognized conditions rather than truly new cases, detecting these conditions early is still beneficial because it allows for timely intervention.^4^ Moreover, successful early detection depends on the active involvement of primary care teams, integration with electronic health records, and adapting tools to suit diverse populations. Involving care partners can improve detection efforts, but the systems must also function effectively when a care partner is unavailable.^2,4^

There is an urgent need for policy advocacy to support and expand access to AWVs.^5,7^ Tzeng et al^3^ demonstrated that a small percentage of eligible older adults used these visits, with even lower participation rates among members of racial and ethnic minority groups, such as Black and Hispanic individuals, and those in rural areas. Integrating AWVs into standard care and ensuring equitable access across demographic groups, particularly underserved populations, is crucial. Policy makers should consider increasing funding, enhancing outreach, and training health care professionals to use these AWVs for the effective early detection of dementia and other age-related conditions.^5^ Tailoring national dementia policies to address specific gaps and strengths can further enhance early detection efforts, aligning with global best practices.^7^

Future research should focus on refining AWV protocols, incorporating detailed patient and caregiver information, and exploring the roles of health care professionals to ensure these visits are both practical and equitable in improving dementia care. By aligning policy with evidence from studies such as this, we can make significant strides in early detection and ultimately transform dementia care for our aging societies.^5,7^

In sum, Medicare AWVs provide a crucial opportunity to detect cognitive decline early, potentially improving care along the pathway to dementia. Success depends on refining protocols, ensuring equitable access, and sustaining investment in research and health care infrastructure. The US can lead this global challenge by integrating early detection into standard care, setting a precedent for proactive, compassionate care. Thoughtful policy and innovative research are essential for a more resilient and responsive approach to dementia care. The time to act is now, as the global increase in dementia cases demands urgent, proactive responses from health care systems.
